# The IKK Inhibitor Bay 11-7082 Induces Cell Death Independent from Inhibition of Activation of NFκB Transcription Factors

**DOI:** 10.1371/journal.pone.0059292

**Published:** 2013-03-20

**Authors:** Hilka Rauert-Wunderlich, Daniela Siegmund, Eduard Maier, Tina Giner, Ralf C. Bargou, Harald Wajant, Thorsten Stühmer

**Affiliations:** 1 Division of Molecular Internal Medicine, Department of Internal Medicine II, University of Würzburg, Würzburg, Germany; 2 Department of Internal Medicine II, Comprehensive Cancer Center Mainfranken, University of Würzburg, Würzburg, Germany; 3 Department of Dermatology, University of Würzburg, Würzburg, Germany; Johns Hopkins School of Medicine, United States of America

## Abstract

Multiple myeloma (MM) displays an NFκB activity-related gene expression signature and about 20% of primary MM samples harbor genetic alterations conducive to intrinsic NFκB signaling activation. The relevance of blocking the classical versus the alternative NFκB signaling pathway and the molecular execution mechanisms involved, however, are still poorly understood. Here, we comparatively tested NFκB activity abrogation through TPCA-1 (an IKK2 inhibitor), BAY 11-7082 (an IKK inhibitor poorly selective for IKK1 and IKK2), and MLN4924 (an NEDD8 activating enzyme (NAE)-inhibitor), and analyzed their anti-MM activity. Whereas TPCA-1 interfered selectively with activation of the classical NFκB pathway, the other two compounds inhibited classical and alternative NFκB signaling without significant discrimination. Noteworthy, whereas TPCA-1 and MLN4924 elicited rather mild anti-MM effects with slight to moderate cell death induction after 1 day BAY 11-7082 was uniformly highly toxic to MM cell lines and primary MM cells. Treatment with BAY 11-7082 induced rapid cell swelling and its initial effects were blocked by necrostatin-1 or the ROS scavenger BHA, but a lasting protective effect was not achieved even with additional blockade of caspases. Because MLN4924 inhibits the alternative NFκB pathway downstream of IKK1 at the level of p100 processing, the quite discordant effects between MLN4924 and BAY 11-7082 must thus be due to blockade of IKK1-mediated NFκB-independent necrosis-inhibitory functions or represent an off-target effect of BAY 11-7082. In accordance with the latter, we further observed that concomitant knockdown of IKK1 and IKK2 did not have any major short-term adverse effect on the viability of MM cells.

## Introduction

Transcription factors of the nuclear factor of kappa light polypeptide gene enhancer in B cells (NFκB) family are ubiquitously expressed and activated by a variety of stimuli including proinflammatory cytokines and environmental stressors [1]. NFκBs control the transcription of hundreds of genes, often encoding for proteins involved in immune regulation but which are also important for cell survival, differentiation and proliferation of non-immune cells [1]. Accordingly, aberrant activity of NFκBs plays a pivotal role in many diseases of the immune system but has also been implicated in aspects of tumor development and metastasis [2], [3]. NFκBs elicit protumoral effects by driving illegitimate gene expression in malignant cells but they are also involved in the maintenance and activity of a tumor promoting microenvironment consisting of, for example, reactive fibroblasts and infiltrating immune cells [4]. NFκB inhibition is therefore considered an attractive option for the development of novel tumor therapies and the antitumoral effects of some established anti-cancer drugs are possibly directly or indirectly related to inhibition of NFκBs [5].

NFκBs are homo- or heterodimers of the structurally related subunits p65 (RelA), RelB, cRel, p50 and p52. The latter two are initially expressed as precursor proteins (p105/NFκB1 and p100/NFκB2) that contain a C-terminal autoinhibitory domain [1]–[3]. In non-stimulated cells, masking of the nuclear localization sequence (NLS) of NFκBs retains them in the cytoplasm. This is achieved either through formation of a ternary complex with proteins of the inhibitor of κB (IκB) family or in the case of p100-containing NFκBs by intramolecular interaction of the NLS with the inhibitory domain of p100 [1]. The two structural modes of NFκB inhibition are related to two prototypic NFκB-activating signaling pathways triggering demasking of the NLS by degradation of IκBs (classical NFκB pathway) or by limited processing of p100 to p52 (alternative NFκB pathway). The classical pathway is stimulated by a wide variety of inducers and typically involves the following, partly overlapping steps: i) stimulus-induced oligomerization of E3 ligases of the tumor necrosis factor (TRAF)- and inhibitor of apoptosis (IAP)-families and non-degradative ubiquitination of various signaling intermediates, ii) recruitment and activation of the IκB kinase (IKK) complex which contains amongst others IKK2 and NEMO, iii) activation of MAP3Ks, iv) MAP3K-mediated phosphorylation and activation of IKK2, v) IKK2-mediated phosphorylation of IκBs, vi) K48 ubiquitination and proteasomal degradation of phospho-IκBs, and finally nuclear translocation of the released NFκB dimer and fine-tuning of its activity by various modifications [1]. Activation of the alternative NFκB pathway is induced by some members of the TNF ligand family, for example TNF-like weak inducer of apoptosis (TWEAK) and B cell activating factor (BAFF), as well as some viral proteins. Stimulation of the alternative NFκB pathway is based upon inhibition of constitutive degradation of the MAP3K NFκB-inducing kinase (NIK) by the concerted action of TRAF2, TRAF3, cIAP1 and cIAP2 and subsequent accumulation of newly synthesized NIK. The latter in turn phosphorylates and activates IKK1 which marks p100 by phosphorylation for limited processing by the proteasome, resulting in p52-containing NFκB dimers which can translocate into the nucleus [1]. Notably, the two NFκB pathways are functionally connected by various mechanisms but nevertheless elicit clearly distinguishable cellular effector programs.

## Materials and Methods

### Ethics Statement

Bone marrow aspirates from MM patients were obtained at the University Hospital of Würzburg, Department of Internal Medicine II, within the frame of diagnostically indicated aspirations and after informed written consent of the respective patients. Permission was granted by the Ethics Committee of the Medical Faculty, University of Würzburg, Würzburg, Germany (ref. no. 18/09).

### Cell Lines, Reagents and Antibodies

HEK293, HT29, and MM.1S cells were obtained from LGC Standards, RPMI8226, AMO1, U266, L363, JJN3, OPM2, and KMS-12-BM cells were obtained from the German Collection of Microorganisms and Cell Cultures, Braunschweig, Germany. INA6 cells were a gift from Martin Gramatzki (Kiel, Germany) [6]. All cell lines were cultivated in RPMI1640 medium (PAA, Cölbe, Germany), containing 10% heat inactivated fetal bovine serum (PAA). INA6 cells were cultivated in medium supplemented with 10 ng/ml recombinant human IL6 (ImmunoTools, Friesoythe, Germany). The isolation and culture of CD138-selected primary MM cells, as well as of primary bone marrow stromal cells (BMSCs) is described in detail in [7]. The Flag-tagged variants of soluble TNF and TWEAK were produced in HEK293 cells and purified by affinity chromatography on anti-Flag mAb M2-agarose (Sigma-Aldrich, Munich, Germany). TPCA-1 and Bay 11-7082 were obtained from Tocris Bioscience (Ellisville, MO, USA) and Calbiochem (Merck, Darmstadt, Germany), respectively. Necrostatin-1 and TRAIL were purchased from Enzo Life Sciences (Lörrach, Germany), and z-VAD-fmk from Bachem (Weil am Rhein, Germany). MLN4924 was obtained from Active Biochem (Maplewood, NJ, USA), butylhydroxyanisole, cycloheximide and propidium iodide were from Sigma-Aldrich. Suppliers of antibodies: NIK (4994) and phospho-IκBα (2859): Cell Signaling Technology (Frankfurt am Main, Germany); p100 (05-361): Merck Millipore (Darmstadt, Germany); tubulin (MS-581-P): Dunn Labortechnik (Asbach, Germany); IκBα (sc847) and p65 (sc109): Santa Cruz Biotechnology (Heidelberg, Germany). Annexin V-FITC (31490013) was purchased from ImmunoTools (Frisoythe, Germany).

### ELISA

Cells were seeded in triplicate in 96-well plates, treated for 30 min with the indicated inhibitors and subsequently stimulated with recombinant TNF. Supernatants were collected and cleared by centrifugation, and the IL8 content was determined with a commercial ELISA kit (BD Biosciences, Heidelberg, Germany) according to the manufacturer’s instructions.

### Western Blotting

Cells were washed once with PBS and lysed in 4× Laemmli sample buffer supplemented with complete protease inhibitor (Roche, Mannheim, Germany) and phosphatase inhibitor cocktails II and III (Sigma-Aldrich). Samples were subjected to sonification, heated to 96°C for 5 min and used for SDS-PAGE. Tansfer to nitrocellulose membranes was performed by wet-blotting, blots were incubated with TBS or PBS containing 0.1% Tween 20 and 5% milk powder prior to sequential incubation with primary antibodies and appropriate horseradish peroxidase-conjugated secondary antibodies (Dako (Hamburg, Germany) and Cell Signaling Technology). For visualization of antigen-antibody complexes the ECL Western Blotting detection system from Pierce, Thermo Fisher Scientific (Bonn, Germany) was used.

### Determination of Cellular Viability

Multiple myeloma cells were seeded in 96-well plates and stimulated with the reagents as described in the figure legends. Metabolically active cells were finally quantified using a standard MTT assay protocol. Cellular viability was calculated from the percentage of the viability of untreated cells (100%) and background staining of completely dead cells (0%). The latter was achieved through a toxic mixture of CD95L, TRAIL, cycloheximide and Bay 11-7082. The alamarBlue viability assay was performed according to the manufacturer's instructions (Morphosys, Martinsried, Germany) on between 25,000 (L363) or 40,000 (JJN3, MM.1S) transfected and purified cells per well (96-well plates) seeded one day after transfection and measured between 3 and 6 days post-transfection.

### Flow Cytometry

Cells were stimulated as described in the figure legends and then washed with cold annexin V buffer (10 mM HEPES, 140 mM NaCl, 2.5 mM CaCl_2_, pH 7.4). Cells were resuspended in 50 µl annexin V buffer supplemented with 1 µl annexin V-FITC and 1 µl propidium iodide solution (1 mg/ml). After 30 min incubation on ice in the dark 100 µl annexin V buffer was added to the cell suspension which was then analyzed using a FACSCalibur (BD Biosciences). Primary MM cells in co-culture with bone marrow stromal cells were carefully dislodged by pipetting, washed with PBS and incubated with annexin V-FITC/propidium iodide solution for 15 min prior to flow cytometric analysis.

### Brightfield Microscopy

For the time lapse experiments 50,000 cells were stimulated in 100 µl medium as indicated and transferred to a chamber slide with a cover slip for microscopy. With heating lamps and a heat sensor the temperature was held constant at 37°C and pictures were captured using a Leica DM IL microscope (Wetzlar, Germany). The pictures of differentially treated cells were captured using an EVOS xl microscope (AMG, Bothell, WA, USA).

### Immunofluorescence Microscopy

HT29 cells (40,000) were seeded on 8-chamber glass slides (BD Bioscience). After adherence, cells were either pretreated for 30 min with Bay 11-7082 (30 µM) or TPCA-1 (20 µM), or remained untreated. Subsequently, cells were stimulated for 1 hour with TNF (100 ng/ml) and stained for immunofluorescence analysis as described [8] using anti-p65 (Santa Cruz Biotechnology) and Cy-3-labeled anti-rabbit (Dianova, Hamburg, Germany) antibodies. Samples were imaged using an EVOS fl microscope (AMG). Mean nuclear and cytoplasmic fluorescence intensities were determined with the ImageJ software to calculate the ratios of nuclear to cytoplasmic fluorescence.

### Statistical Analysis

Error bars shown in the various graphs represent standard error of the mean (SEM). Statistical analyses were performed using GraphPad Prism 5 (GraphPad Software, Inc., La Jolla, CA, USA), with details described in the legends to the respective figures. All data shown are representative for at least two independent experiments.

### Sequences

Stealth siRNAs were purchased from Life Technologies (Darmstadt, Germany) and used at a final concentration of 3 µM (per duplex) in the electroporation mix. The nucleotide sequences (sense direction) for the stealth siRNAs were: GCAUUCAGCUUGACUUGGAGAGAUA (against human IKK1), CGAACUGAGGGUGACAGUCAGGAAA (against human IKK2), and AUUCUCCGAACGUGUCACGUAGCUA (nonspecific). Electroporation conditions, and subsequent purification of strongly transfected cells are described in detail in [9].

## Results and Discussion

### Bay 11-7082 is Highly Toxic at Concentrations of Effective IKK Inhibition

**Figure 1:**
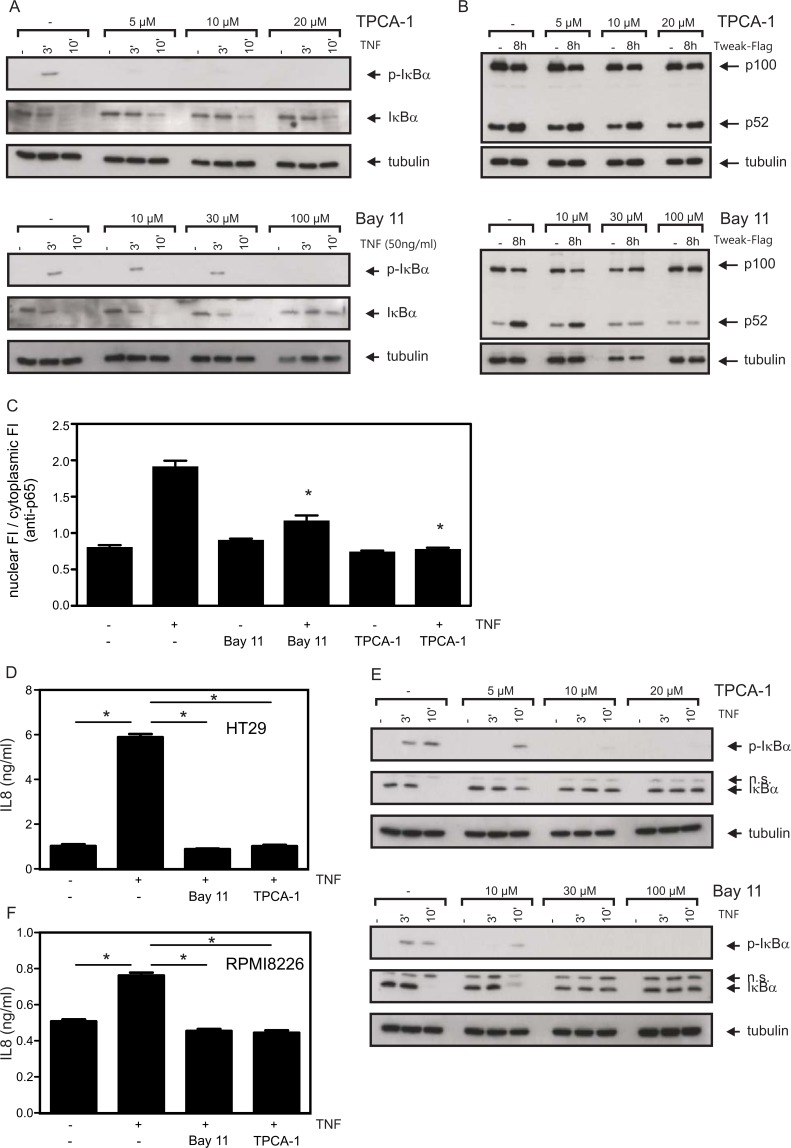
Functional titration of IKK inhibitors. (A,B) HT29 cells were pretreated for 1 h with the indicated concentrations of TPCA-1 and Bay 11-7082 and were subsequently challenged with TNF (50 ng/ml) for 3 and 10 min (A) or with Flag-TWEAK (200 ng/ml) for 8 hours (B). Total cell lysates were finally analyzed by Western blotting with respect to TNF-induced phosphorylation and degradation of IκBα (A) and TWEAK-induced p100 processing (B). (C) HT29 cells (40,000/chamber) were grown on glass slides and were pretreated with Bay 11-7082 (30 µM) or TPCA-1 (20 µM) for 30 min. Cells were then challenged with 100 ng/ml TNF for 1 h. After immunofluorescence staining for p65, the ratio of nuclear to cytoplasmic fluorescence intensity (FI) was determined. Data shown corresponds to 95–111 analyzed cells per experimental condition derived from a total of four independent experiments. (D) HT29 cells (20,000/well, 96 well-plate, triplicate values) were pretreated with the IKK inhibitors Bay 11-7082 (30 µM) or TPCA-1 (20 µM) for 30 min and were then stimulated with 100 ng/ml TNF for 6 h. The IL8 content of supernatants was subsequently determined by ELISA. To minimize the background signal related to constitutive IL8 production, medium was changed prior to inhibitor treatment. (E) The effects of TPCA-1 and Bay 11-7082 on TNF-induced phosphorylation and degradation of IκBα were analyzed in KMS-12-BM myeloma cells as described under “A”; n.s. = non specific. (F) Equivalent analysis on TNF-induced IL8 production as described in “D” using the RPMI8226 MM cell line. For statistical analysis of data shown in C, D and F one-way ANOVA with a Tukey post-test was performed. Asterisks indicate p-values≤0.01.

To differentiate between contributions of the classical and the alternative NFκB pathway for the survival of multiple myeloma (MM) cells, we used 2-[(aminocarbonyl)amino]-5-(4-fluorophenyl)-3-thiophenecarboxamide (TPCA-1), an inhibitor with high preference for IKK2 over IKK1, and Bay 11-7082, an inhibitor with comparable activity against both IKKs. Initially, we used the colon carcinoma cell line HT29 to monitor TNF-induced phosphorylation and degradation of IκBα, two events crucially dependent on IKK2 activity, and of TWEAK-induced processing of p100, which is mediated by IKK1, in order to determine functionally active inhibitor concentrations. Treatment with 10 µM TPCA-1 was sufficient to largely block TNF-induced phosphorylation and degradation of IκBα without showing an inhibitory effect on TWEAK-stimulated p100 processing (Fig. 1A,B). Some reduction of p100 expression levels without concomitant increase in p52 was observed after TPCA-1 treatment (Fig. 1B), which is likely due to the fact that *NFKB2* (the gene encoding p100) is a target gene of the classical NFκB pathway [10]. Bay 11-7082 inhibited both TWEAK-induced p100 processing and TNF-induced phosphorylation and degradation of IκBα. Significant blockade of both NFκB pathways was achieved at concentrations between 30 to 100 µM of Bay 11-7082 (Fig. 1A,B). In accordance with their inhibitory effect on TNF-induced degradation of IκBα, p65 nuclear translocation and upregulation of IL8, which is encoded by a target gene of the classical NFκB pathway, were efficiently blocked by TPCA-1 and Bay 11-7082 (Fig. 1C,D). Both inhibitors displayed a similar dose dependency on TNF-induced phosphorylation and degradation of IκBα in the MM cell line KMS-12-BM and also prevented upregulation of IL8 in the MM cell line RPMI8226 (Fig. 1E,F).

**Figure 2:**
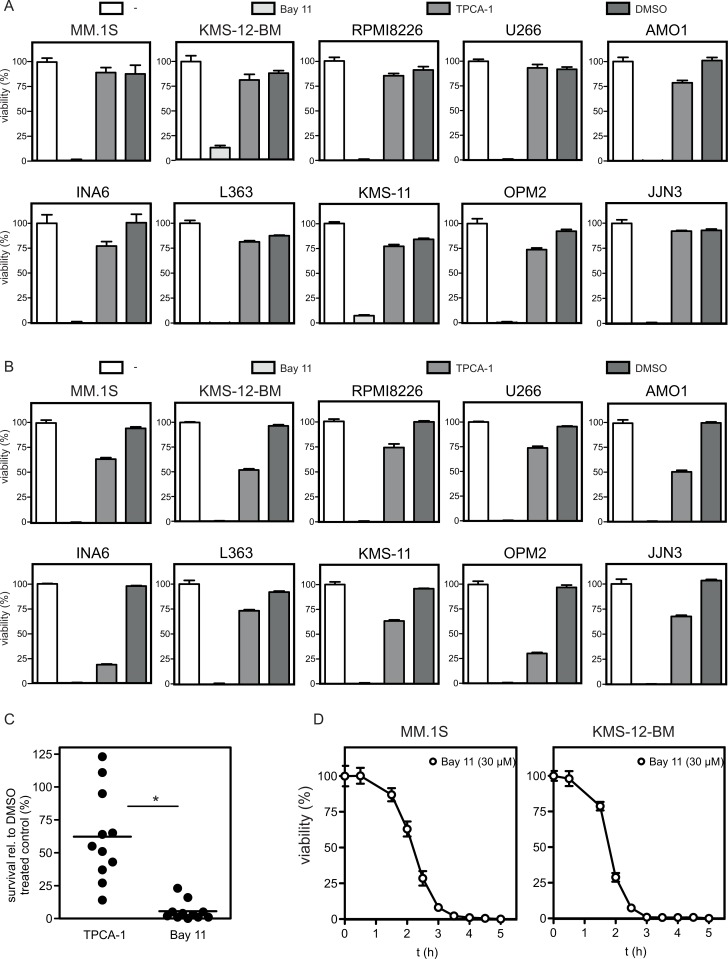
Effects of IKK inhibitors on MM cell viability. (A,B) MM cell lines were challenged with either TPCA-1 (20 µM) or Bay 11-7082 (30 µM) for 4 h (A) and 18 h, respectively (B), and analyzed for viability using the MTT assay. (C) Primary MM samples (n = 11) in co-culture with BMSCs were incubated with either TPCA-1 (20 µM) or Bay 11-7082 (30 µM) for 3 days and cell death was determined by annexin V-FITC/PI staining and flow cytometry (relative values with respect to the DMSO-treated controls shown; horizontal lines indicate the mean). Data were analyzed using a two-tailed, paired t-test. Asterisk indicates a p*-*value <0.01. (D) MM.1S and KMS-12-BM cells were challenged with 30 µM Bay 11-7082 for the indicated time. Triplicate values were taken and cellular viability was determined using the MTT assay.

We next analyzed the effects of TPCA-1 and Bay 11-7082 on MM cell viability in a panel of 10 MM cell lines as well as in primary MM samples. Treatment with TPCA-1 [20 µM] had virtually no effect after 4 h and entailed moderate viability decreases (0–50%) after 18 h in most MM cell lines (up to 75% in INA6 and OPM2) (Fig. 2A,B). Freshly isolated primary MM cells (n = 11) kept in co-culture with bone marrow stromal cells and exposed to TPCA-1 [20 µM] for 3 days showed cell death induction (annexin V positivity) to quite variable extent, with a median survival of 61% relative to DMSO-treated controls (Fig. 2C). Of note, Bay 11-7082 [30 µM] induced complete cell death (<10% of control) in all cell lines as well as in the large majority of primary MM samples (Fig. 2A,B,C). A refined analysis of cell death induction with 30 µM Bay 11-7082 in KMS-12-BM and MM.1S MM cells revealed very rapid kinetics. Reduced viability was already detectable after 1 h and the complete effect was typically reached within 2–4 h (Fig. 2D).

### Cell Death Induction by Bay 11-7082 Involves Necrosis-related Mechanisms

**Figure 3:**
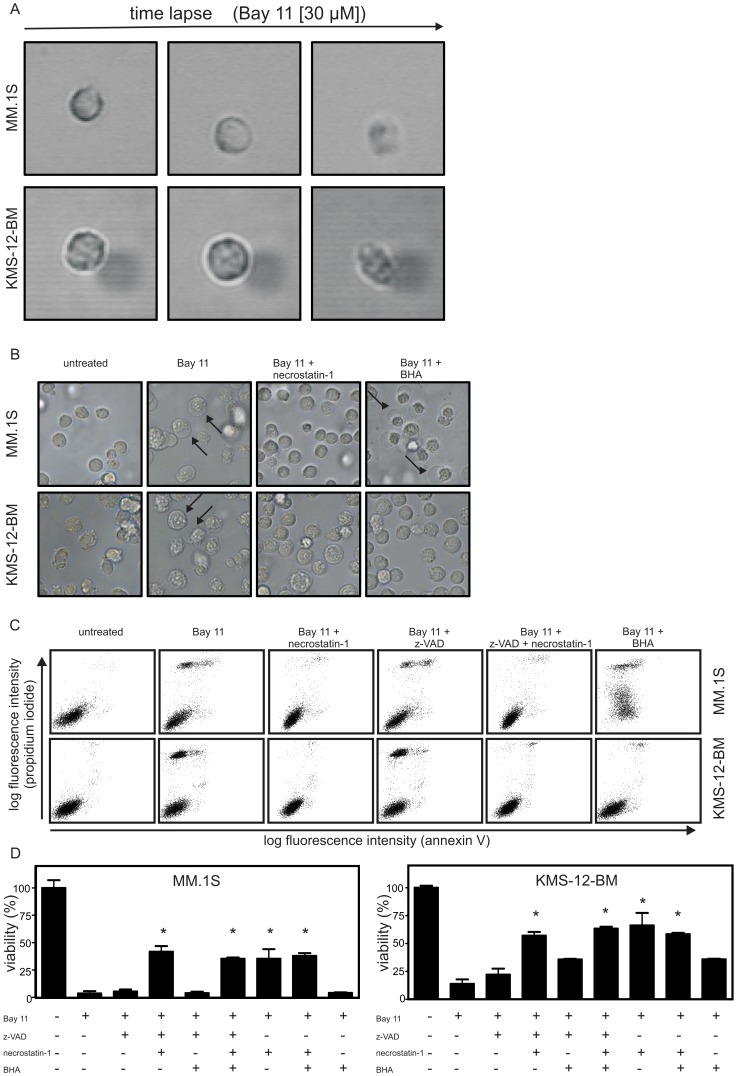
The mechanism of Bay 11-7082-induced MM cell death involves necrosis. (A) MM.1S and KMS-12-BM cells were treated with 30 µM Bay 11-7082 and analyzed by time-lapse video microscopy. Pictures shown represent typical stages of cells undergoing Bay 11-7082-induced cell death within 3 h. (B) Cells were pretreated for 30 min with BHA (50 µM), necrostatin-1 (90 µM) or remained untreated and were then challenged with 30 µM Bay 11-7082 for 2 h. Cells were finally photographed (B, arrows indicate swollen cells and the plasma membrane). (C) MM cells were either left untreated or pretreated for 1 h with BHA (50 µM), necrostatin-1 (90 µM), z-VAD-fmk (100 µM) or both necrostatin-1 and z-VAD-fmk. Cells were then challenged with 15 µM Bay 11-7082 for 1 h (KMS-12-BM) or 2 h (MM.1S) followed by annexin V-FITC/PI staining and FACS analysis. (D) MM.1S and KMS-12-BM cells were pretreated in triplicates for 30 min with the indicated combinations of BHA (50 µM), necrostatin-1 (90 µM) and z-VAD-fmk (100 µM), exposed for 2 h to 30 µM Bay 11-7082 and then analyzed for viability using the MTT assay. For statistical analysis a one-way ANOVA with a Tukey post-test was performed. Experimental settings that display significant protection against Bay 11-7082 induced cell death (p-values≤0.01) are indicated by asterisks.

Treatment of MM cells with Bay 11-7082 induced rapid swelling and subsequent desintegration of the cellular body, which are typical signs of necrosis (Fig. 3A). Necrosis-related forms of cell death often involve generation of reactive oxygen species (ROS) and engage the kinase receptor interacting protein-1 (RIP1) [11]. To evaluate whether these molecules also play a role in Bay 11-7082-induced cell death, we used the antioxidant butylhydroxyanisole (BHA) and necrostatin-1, which inhibits the kinase activity of RIP1 [12]. Both compounds were initially able to block the detrimental effects of 30 µM Bay 11-7082 in KMS-12-BM cells (Fig. 3B–D) but were still unable to rescue the cells for extended periods of time (Fig. 3D). However, BHA was ineffective from the outset in MM.1S cells (Fig. 3B–D). The failure of necrostatin-1 to rescue MM cells after prolonged incubation with Bay 11-7082 was not due to concomitant activation of caspases, because virtually no activation of caspases was observed in cells challenged with Bay 11-7082 (data not shown) and the pan-caspase inhibitor z-VAD-fmk did not improve the protective effect of necrostatin-1 (Fig. 3C,D).

### The NAE-inhibitor MLN4924 Efficiently Inhibits Cytokine-induced Activation of the Classical and Alternative NFκB Pathways Downstream of IKKs but is Less Cytotoxic than Bay 11-7082

**Figure 4:**
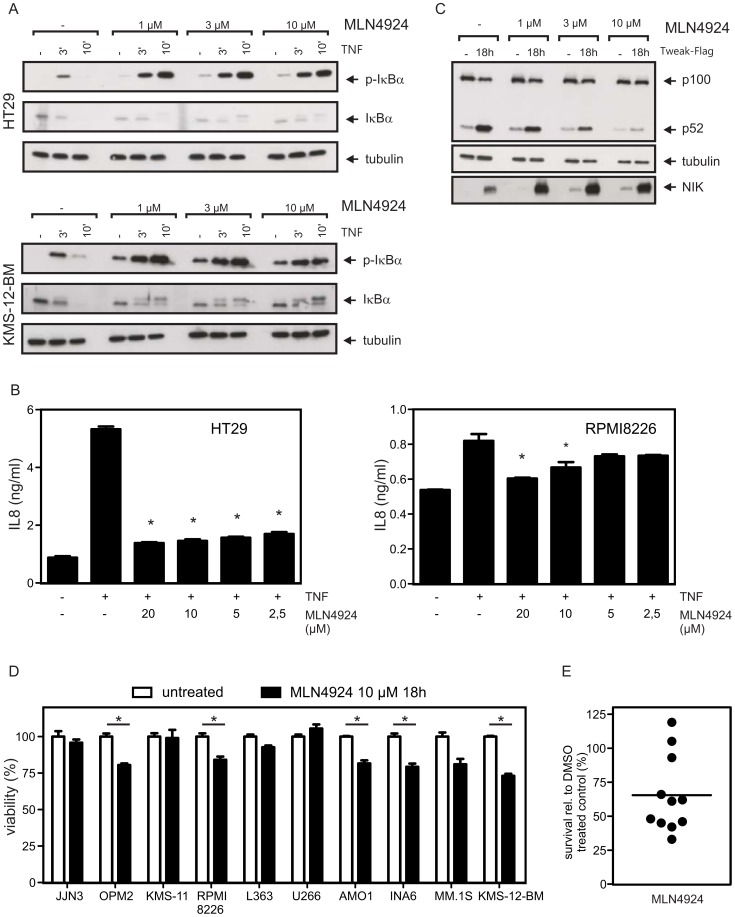
Functional titration of MLN4924 and effects on MM cell viability. (A) HT29 and KMS-12-BM cells were pretreated for 1 h with the indicated concentrations of MLN4924 and challenged with TNF (50 ng/ml) for 3 and 10 min. Phosphorylation and degradation of IκBα were subsequently analyzed by Western blotting of total cell lysates. (B) HT29 cells (20,000/well, 96 well-plate, triplicate values) and RPMI8226 cells (50,000/well, 96 well-plate, triplicate values) were pretreated for 30 min with different concentrations of MLN4924. Cells were then stimulated with 100 ng/ml TNF for 6 h and the IL8 content of supernatants was determined by ELISA. For statistical analysis a one-way ANOVA with a Tukey post-test was performed. Groups showing significant inhibition (p-values≤0.01) of TNF-induced IL8 production are indicated by asterisks. (C) HT29 cells were pretreated for 1 h with the indicated concentrations of MLN4924 and then stimulated with Flag-TWEAK (200 ng/ml) for 18 h. The levels of p52, p100 and NIK were analyzed by Western blotting of total cell lysates. (D) MM cell lines were treated for 18 h with 10 µM MLN4924 and assayed for viability using the MTT assay. Significant (unpaired, two-tailed t-test, p-values≤0.01) induction of cell death is highlighted by asterisks. (E) Primary MM samples (n = 11) in co-culture with BMSCs were treated with 10 µM MLN4924 for 3 days and cell death was assessed by annexin V-FITC/PI staining and FACS analysis.

The much stronger cytotoxic effect of Bay 11-7082 compared to the IKK2-specific inhibitor TPCA-1 could indicate that NFκBs regulated by the alternative NFκB pathway are the most crucial ones for MM cell survival but could also be a consequence of either NFκB-independent functions of IKK1 or of off-target effects. We therefore chose an alternative approach to analyze the cytotoxic effects of inhibition of the classical and alternative pathways at a level downstream of IKK1 and IKK2. For this purpose, we used the NEDD8 activating enzyme (NAE)-inhibitor MLN4924 [13]. Degradation of IκBα in response to its phosphorylation by IKK2, as well as p100 processing in response to its phosphorylation by IKK1 require a βTrCP-containing E3 ubiquitin ligase complex of the SCF (SKP 1 – cullin 1 – F-box protein) family [14], [15]. Modification of the Cul-1 component of the SCF^βTrCP^ complex with NEDD8 in turn is required for both ubiquitination of IκBα and p100 processing [1], [14], [15]. Accordingly, MLN4924 inhibited TNF-induced degradation of IκBα and IL8 production without blocking IκBα phosphorylation (Fig. 4A,B). Likewise, treatment with MLN4924 strongly reduced TWEAK-induced p100 processing and led to NIK accumulation (Fig. 4C). At concentrations where the inhibitory effect of MLN4924 on NFκB signaling was comparable or even stronger than those of Bay 11-7082, this compound was substantially less toxic on MM cells from either cell lines or primary origin (Fig. 4D,E). Thus, the cytotoxic effects of Bay 11-7082 in MM cells are not related to inhibition of NFκBs and must therefore be caused by interference with NFκB-independent functions of IKK1 or by off-target effects. Indeed, there is also evidence for NFκB-unrelated cell death induction by Bay 11-7082 from studies with Ewing’s sarcoma tumor cell lines and precursor-B acute lymphoblastic leukaemia cell lines and blasts [16], [17]. Moreover, a recent study by Lee et al. shows that Bay 11-7082 functions as an inhibitor of multiple proinflammatory signaling pathways resulting in the activation of AP-1, STAT-1 and IRF-3 [18]. However, given the drug’s strong propensity to initiate cell necrosis it remains possible that some of the anti-inflammatory effects described in this report represent secondary consequences of cell disintegration rather than a direct inhibitory effect of Bay 11-7082. Of note, although TPCA-1 as well as MLN4924 did show dose-dependent impairment of MM cell viability and induction of MM cell death, at concentrations sufficient to block either the classical (TPCA-1, 20 µM) or the classical and alternative pathways (MLN4924, 10 µM) these compounds did not differ substantially in their capacity to elicit the respective effects. If anything, the neddylation inhibitor appeared even somewhat less toxic than TPCA-1 in short-term (18 h) in vitro treatments of MM cells (Fig. 2B vs. 4D), whereas their effects on the survival of primary MM cells co-cultured with BMSCs and exposed to either 20 µM TPCA-1 or 10 µM MLN4924 for 3 days were broadly similar (Fig. 2C vs. 4E). This result did not suggest that the concomitant inhibition of both NFκB pathways was superior in its anti-myeloma efficacy to using the IKK2 inhibitor alone.

### Knockdown of IKKs did not Adversely Affect MM Cell Growth and Survival in Shorter Time Frames

**Figure 5:**
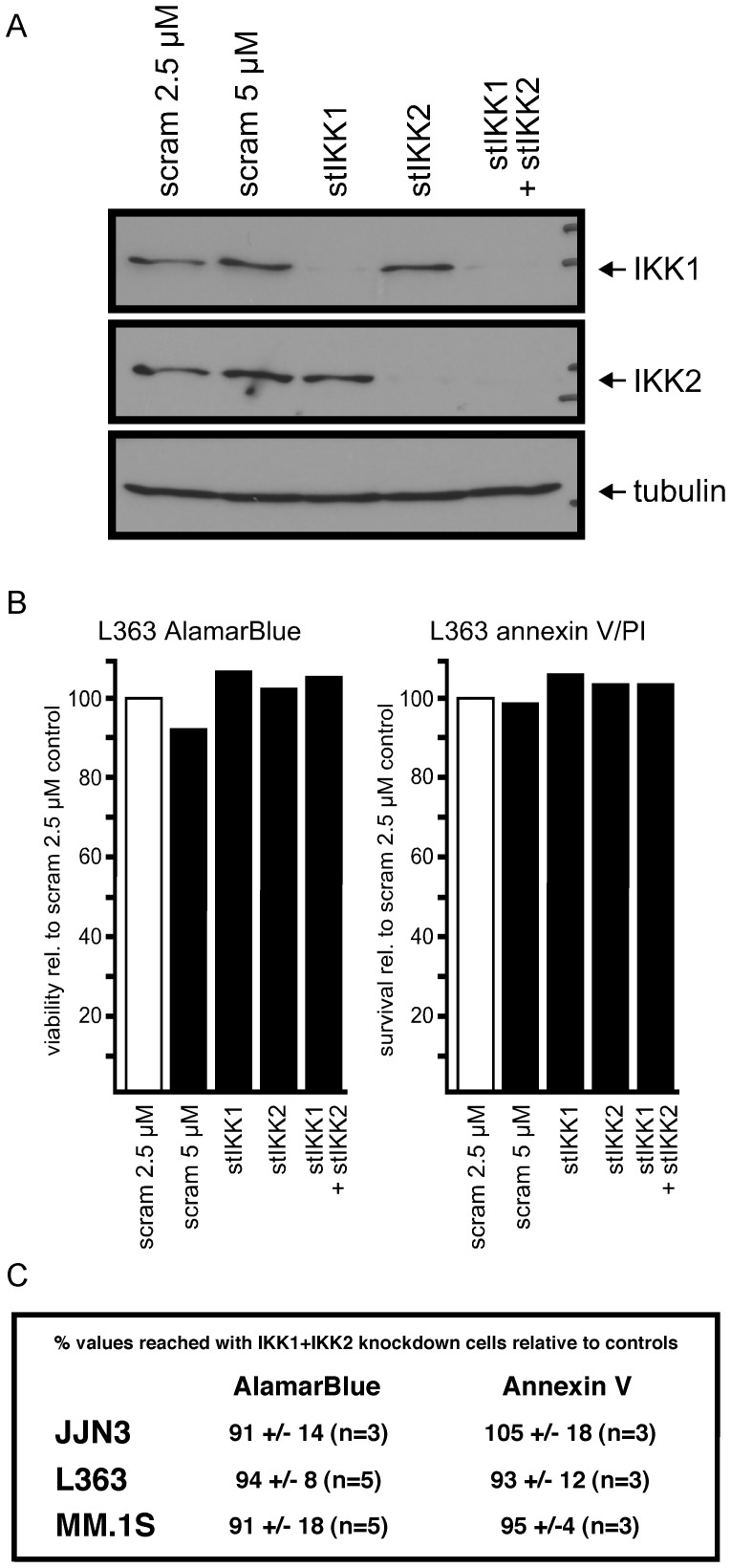
Knockdown of IKK1, IKK2 or both in MM cells. (A) Western blots showing depletion of IKK1 and IKK2 in L363 cells after single or combined transfection with stealth siRNAs for the respective targets. Scram denotes non-specific stealth siRNA control. Samples prepared at day 2 post-electroporation from cells co-transfected with an expression plasmid for CD4Δ and purified by CD4 microbead selection of strongly transfected cells. (B) Viability (AlamarBlue, left panel) and survival (annexin V, right panel) assays measured at day 4 post-electroporation for the sample preparations shown in “A”. All values calculated relative to the measurement obtained for the non-specific siRNA control at 2.5 µM concentration (i.e. the concentration present in single stealth siRNA transfections). (C) Overview of the viability and survival effects of IKK1 and IKK2 knockdown in MM cell lines JJN3, L363 and MM.1S. Mean values (calculated with respect to the respective non-specific siRNA controls) and standard deviations shown.

In order to probe the effects of NFκB pathway abrogation by other than pharmacological means we also used siRNAs against the IKKs in order to attenuate intrinsic activities of the NFκB pathways in MM cell lines JJN3, L363 and MM.1S. Cells were electroporated with stealth siRNA against either IKK1 (stIKK1) or IKK2 (stIKK2), or with a mixture of both. A non-specific siRNA duplex (scram) served as transfection control. Additionally, expression plasmids for EGFP (to monitor transfection efficiency) and CD4Δ (to permit purification and further culture of only the strongly transfected cells [9]) were co-transfected. At concentrations of 2.5 µM in the electroporation mix the siRNAs led to profound depletion of IKK1 and/or IKK2 (exemplarily shown at day 2 post-transfection for L363 cells; Fig. 5A) lasting for at least 5 days (not shown). However, neither general viability, as determined by the alamarBlue metabolic colorimetric assay, nor explicit cell survival, as determined by staining with annexin V-FITC and propidium iodide followed by flow cytometric analysis, were substantially impaired in any of these cell lines (an exemplary measurement of the purified transfected cells at day 4 post-transfection for both of these assays is shown for L363 cells in Fig. 5B). MM cells with single knockdown of either IKK1 or IKK2 showed very little digression from the values for the control transfections (measured at days 3, 4 and 5 post-transfection; Fig. 5B and data not shown), and even though the double knockdown of IKK1 and IKK2 tended to entail slightly lower values for viability/survival in some experiments, on average less than 10% decline from control values was noted (Fig. 5C for experimental values at day 4 post-transfection, and data not shown).

Even though the amount of NFκB activity in MM cells does not generally appear to exceed levels normally seen in plasma cells [19], the presence of genetic lesions conducive to the generation or increase of cell autonomous activation of the NFκB system in a significant number of MM samples strongly implies a functional role in the molecular aetiology of this disease [19]–[22]. A number of IKK2 inhibitors have been found toxic to MM cells (e.g. [23]–[27]) although the described effects vary considerably, and it has recently been suggested that dual inhibition of the alternative and classical pathways is required to elicit MM cell death, as shown with the Rel protein inhibitor PBS-1086 [28]. We observed a striking discordance in the anti-MM effects of the different pharmacological NFκB-pathway inhibitors tested, but also vis-à-vis IKK1 and 2 depletion by siRNA-mediated knockdown. Whereas in the case of Bay 11-7082 its high anti-MM toxicity is mostly attributable to necrosis induction, it remains surprising that the reasonably extensive IKK double knockdown failed to even mildly emulate the more moderate toxicity seen with either TPCA-1 or the neddylation inhibitor MLN4924, especially as the MM cell lines used are known to harbor mutations that activate the NFκB system [19], [20]. Whether and in which genetic background blockade of the NFκB system is a suitable therapeutic option to induce MM cell apoptosis (as opposed to growth inhibition relying on long-term systemic drug treatment) thus remains an open question. A more detailed study in MM cells addressing the consequences of molecular ablation of the actual transcription factors themselves should provide further clues towards an answer.
